# Lateralization of Motor Cortex Excitability in Stroke Patients during Action Observation: A TMS Study

**DOI:** 10.1155/2014/251041

**Published:** 2014-04-14

**Authors:** Mattia Marangon, Konstantinos Priftis, Marta Fedeli, Stefano Masiero, Paolo Tonin, Francesco Piccione

**Affiliations:** ^1^Department of General Psychology, University of Padova, Via Venezia, 8-35131 Padova, Italy; ^2^Fondazione Ospedale San Camillo I.R.C.C.S., Via Alberoni 70, 30126 Venezia-Lido, Italy; ^3^Department of Rehabilitation Medicine, University of Padova, Via Giustiniani 1, 35128 Padova, Italy

## Abstract

Action observation activates the same motor areas as those involved in the performance of the observed actions and promotes functional recovery following stroke. Movement observation is now considered a promising tool for motor rehabilitation, by allowing patients to train their motor functions when voluntary movement is partially impaired. We asked chronic-stroke patients, affected by either left (LHD) or right hemisphere (RHD) lesions, to observe either a left or right hand, while grasping a small target (eliciting a precision grip) or a large target (eliciting a whole hand grasp directed towards a target object). To better understand the effects of action observation on damaged motor circuits, we used transcranial magnetic stimulation (TMS) to induce motor evoked potentials (MEP) from two muscles of the unaffected hand in 10 completely hemiplegic participants. Results revealed that LHD patients showed MEP facilitation on the right (contralesional) M1 during action observation of hand-object interactions. In contrast, results showed no facilitation of the left (contralesional) M1 in RHD patients. Our results confirm that action observation might have a positive influence on the recovery of motor functions after stroke. Activating the motor system by means of action observation might provide a mechanism for improving function, at least in LHD patients.

## 1. Introduction

Stroke results in irreversible brain damage, in which the most common neurological impairment is contralesional partial weakness, reflecting a reduced ability of the affected patient to activate spinal motoneurons voluntarily. Chronic motor problems cause patients difficulties in using their hand, for example, in everyday activities. Other impairments often accompany hemiparesis, such as hemianesthesia, hemianopsia, aphasia, and dysarthria [1–3]. Only about 40% of stroke survivors recover completely, whereas the remaining 60% of stroke survivors have permanent sensory and/or motor impairments that significantly disrupt their ability to participate in home activities and community life [4]. Despite substantial advances in the development of effective training protocols, aiming to enhance function of the affected hand after stroke, functional recovery is usually incomplete. Thus, most stroke survivors experience long-term motor disability.

Within this context, observation of normal motor performance may assist the recovery of patients' hampered hand movements following stroke, simply by activating the affected cortical motor network in a way similar to that during movement execution by the patient. Thereby, movement observation might enhance beneficial changes within this network [5–7].

Evidence from functional magnetic resonance imaging (fMRI) studies has suggested that the neural network involved in action execution overlaps extensively with that activated when actions are observed [8, 9]. This shared neural network includes the premotor cortex, the supplementary motor area (SMA), the inferior parietal lobule, the cingulated gyrus, and the cerebellum [10].

In the abovementioned, shared neural network, the primary motor cortex (M1) plays a crucial role in receiving inputs from all the areas composing the network; thus, M1 contributes to many different motor representations [9]. The involvement of M1 in action observation has been confirmed by studies in which the technique of transcranial magnetic stimulation has been employed (TMS) [11–15].

Single-pulse TMS induces an involuntary muscle contraction that can be measured with electromyography (motor-evoked potential; MEP). MEPs are used to quantify the motor output resulting from depolarization of cortical neurons through TMS stimulation. By using TMS, Fadiga and colleagues (1995) showed that during the observation of another person's hand actions, TMS-induced MEPs recorded from hand muscles were facilitated. This dynamic modulation of corticospinal excitability (CSE) has been attributed to the activity of an observation-execution matching system, by which visual information concerning an observed action is integrated in the observer's motor system [11].

Motor facilitation during action observation has been replicated in numerous studies and it is now well established that corticospinal (CS) facilitation induced by the observation of grasping movements is topographically attuned to the type of grasp being observed (i.e., precision versus whole-hand grasp). This suggests that motor coding is based on the visual aspects, which characterize an observed movement, regardless of the specific goal of the observed action [12].

Moreover, motor facilitation, measured through TMS-induced motor evoked potentials (MEP), replicates the temporal profile of the observed movement [11–18]; it is modulated by experience [12], and it can be influenced by the specific visual perspective (first-person or third-person perspective) [19]. Behaviourally, the position of the demonstrator (i.e., the person who performs the to-be-observed actions) appears to influence the CS system, with the first-person perspective producing a significantly enhanced MEP in the muscle recruited in the observed action [20–22]. Taken together, these studies provide strong evidence that action observation appears to activate the motor system in a way similar to that during motor execution, by generating an internal representation of action that can be a target for motor relearning.

The question of whether observation of goal-directed movement produces a pattern of facilitation across muscles that is consistent with the observed action is crucial for evaluating its potential as a rehabilitation tool. In this context, action observation is considered as a technique that could increase motor function, by helping stroke patients to relearn to accommodate changes associated with alterations that accompany injuries [7, 13].

Internal motor representations are a key mechanism through which the motor system contributes to observation of actions [23, 24]. Motor representation refers to applying internal models to perceived actions that are used in the planning and execution of one's own actions. This is important for at least two reasons. First, because action observation tasks produce these effects even when others' movements are observed passively [25], they may provide a way of stimulating sensory and motor systems in patients who may be unable to follow detailed instructions or who have significant cognitive impairments in association with their motor impairments. Second, in individuals who can follow instructions, it is possible to have them imagine movements of their own bodies while observing another individual undertaking the very same actions. Combining these two forms of stimulation might augment the amount of activity within sensory and motor areas and increase our ability to prevent maladaptive reorganization within these areas or facilitate reestablishment of preinjury organization of function.

The main goal of the present study was to assess the changes in corticospinal excitability in hand muscles when stroke patients are asked to observe goal-directed hand actions. In addition, we aimed to investigate what stimulation parameters (object size, visual perspective, and biological effector), reflected in CSE, are crucial for rehabilitation purposes.

To this aim, TMS-induced MEPs were recorded, while patients observed videos showing grasping movements either of the right or the left hand. Patients were also shown control videos showing a static hand, close to a target object. We used target objects of different sizes: a small target eliciting a precision grip or a large one eliciting a whole hand grasp. Actions were viewed from a first-person perspective (i.e., the actions were presented from the viewpoint of the observers, as if they were to perform the action by themselves). Rather than simply focusing on a comparison of MEP amplitudes across trials, we calculated the action MEP amplitudes as a ratio of the static MEPs in order to report the modulation in MEP, which were due to the observation of the action* per se*.

## 2. Methods

### 2.1. Participants

Ten right-handed stroke patients volunteered in this study (eight males, two females; mean age: 57; SD: 10 years). All participants were right-handed, according to the 10-item version of the Edinburgh Inventory [26]. All patients were examined at least 6 months after stroke. They gave their written informed consent prior to inclusion, and the study was approved by the research ethics committee (REC) of the Scientific Foundation “San Camillo” according to the Declaration of Helsinki.

We screened and excluded patients older than 80 years and patients with history of substance abuse and/or psychiatric diagnosis, impaired level of consciousness (confusion, stupor, and coma), severe to moderate aphasia, anosognosia, neglect, amnesia, dementia, and depression. Patients with contraindications to TMS, including those patients with metallic implants, history of seizures, and patients under medication, known to alter CSE, were also excluded.

All 10 patients suffered from a first-time unilateral stroke. Five patients were affected by left hemisphere stroke (LHD) and five suffered from a right hemisphere stroke (RHD). The LHD patients group consisted of five males (mean: 60 years; SD = 11.7). Four LHD patients had a right-sided hemiparesis. The group of RHD patients included four males and one female (mean: 57 years; SD = 11.9). Four RHD patients had a left-sided hemiparesis. Because of discomfort with TMS stimulation, the RHD female participant withdrew from the experiment following the procedure implying the determination of the optimal scalp position (OSP). No discomfort or adverse effects during TMS were reported or noticed in any of the other participants.

All patients (LHD, RHD) showed at least a weak capacity to perform finger movements with their affected hand. TMS induced MEPs, recorded in the affected FDI and ADM hand muscles of all participants, showed low amplitudes in the contralesional hands.

Both groups were tested by means of the Barthel index (BI) and of the functional independence measure (FIM). The Barthel index is a 10-item instrument measuring functional independence in personal activities of daily living. The FIM is an 18-item scale for assessing physical and cognitive disability. FIM's items are scored on the level of assistance required for a person to perform activities of daily living (13 items). The FIM also includes measures of communication and social cognition (five items). There are three FIM scores: a total score (FIM total: 18 items), a physical score (FIM physical: 13 items), and a cognitive score (FIM cognitive: five items).  Table 1 summarizes demographic and clinical data of each group.

All patients had undergone intensive physiotherapy before entering in our study. All of them were screened for contraindications to TMS and for recent alcohol and caffeine intake [27].

### 2.2. Task and Apparatus

Participants were seated in a comfortable chair in front of a computer screen with support for the arms and hands. Support for the hands was ad hoc built to allow participants to remain comfortable with their hands in a pronated position throughout the experiment. A custom-designed cushion around the neck was used to ensure participants' comfort and also to limit head movements during the experiment. Participants were instructed to fully relax their muscles and to pay attention to the visual stimuli presented on a 19-inch monitor (resolution: 1280 × 1024 pixels; refresh frequency: 75 Hz), positioned 80 cm in front of them, at eye level. To make them pay attention, patients were told that they would be questioned at the end of the experiment about what they had seen. Three different types of colored videoclips were used as experimental stimuli: (i) a reach-to-grasp action video towards a small object-target with a precision grip; (ii) a reach-to-grasp action video towards a large object with a whole hand grasp; (iii) a static image of a hand with either a small or a large target-object. Each videoclip showed either a left or a right hand, while grasping the target object.

At the beginning of each videoclip, the hand of the demonstrator was shown in a prone position resting on a table with the object-target placed in front of it. After 500 ms, the hand started moving. Each videoclip lasted 3750 ms. The animation effect was obtained by presenting a series of single frames, each lasting 30 ms (resolution 720 × 576 pixels, color depth of 24 bits, and frame rate of 30 fps) except for the first and the last frame, which lasted 500 and 1000 ms, respectively.

Stimulus presentation timing, randomization of stimuli, and TMS triggering were controlled by means of the E-Prime V2.0 software (Psychology Software Tools Inc., Pittsburgh, PA, USA) running on a PC. The experiment consisted of four counterbalanced blocks of three types of videoclips. A total of 96 trials resulted from the combinations of four possible stimuli (small or large objects), two positions for the performed movement (left or right hand), two types of observed movement (precision grip or whole hand grasp), and two TMS delivery times (early, late). The order in which the blocks were presented was randomized across the participants. The two TMS-delivery timings have been selected on the basis of previous literature [16].

### 2.3. Electromyographic and TMS Recording

Electromyographic (EMG) traces were recorded from the first dorsal interosseous (FDI) and abductor digiti minimi (ADM) muscles of either the right or the left hand, by means of 9 mm diameter, Ag-AgCl surface cup electrodes. For the FDI muscle, the active electrode was placed over the muscle belly and the reference electrode over the metacarpophalangeal joint of the index finger. For the ADM muscle, the active electrode was placed over the muscle belly and the reference electrode over the metacarpophalangeal joint of the little finger. Responses were amplified with a Neuroscan amplifier (Neuroscan Amplifier, Compumedics Neuroscan), through filters set at 20 Hz and 2 kHz with a sampling rate of 5 kHz and then recorded by a computer using Neuroscan software (Neuroscan, Compumedics Neuroscan). TMS pulses were delivered using a 70 mm figure-of-eight coil connected to a Magstim Rapid^2^ (Magstim, Whitland, Dyfed, UK) placed over either the left or the right primary motor cortex, contralaterally to the EMG-recorded muscles.

Both hemispheres were searched to find stimulation points eliciting contralateral EMG activity, and the optimal points (OP; defined as the scalp position where a reproducible muscle response was elicited with the lowest stimulation intensity), as well as their resting motor thresholds (MT; defined as the minimum stimulation intensity that produced at least 5 MEPs exceeding 50 *μ*V in 10 trials), were determined separately [24]. Once the optimal point was localized, the site was marked with a red dot to ensure consistent coil positioning. The same experimenter was responsible for holding the coil in place for the entire duration of the experiment.

The intensity of TMS was adjusted to evoke an MEP of approximately 1 mV peak-to-peak in the relaxed right or left FDI and ADM. The hand motor area of the left and right M1 was defined as the point where stimulation evoked the largest MEP from both muscles. In order to prevent contaminations of MEPs by background EMG activity, a window of 100 ms before TMS pulse was used to check for trials with any background activity greater than 100 *μ*V. The coil was placed tangentially to the scalp with the handle pointing backward and laterally at 45° to the sagittal plane, inducing a posterior-anterior current in the brain.

### 2.4. Data Analysis

Data were analyzed offline using the Neuroscan software (Neuroscan, Compumedics Neuroscan) and SPSS 17.0.1 (SPSS Inc., Chicago, IL, USA). Background EMG level prior to TMS was calculated for each trial. Individual mean peak-to-peak amplitudes of MEPs recorded from the FDI and the ADM muscles were calculated separately for each baseline condition, each type of observed movement (grasping small object, grasping large object, and static image), and each TMS delivery timings (early, late). MEPs amplitudes were averaged for each muscle and type of movement observed.

We did not record any reliable MEPs (≥100 *μ*V peak-to peak amplitude) from the impaired (contralesional) hand muscles in the 9 participants. In contrast, TMS induced MEPs, recorded in the unaffected (ipsilesional) FDI and ADM hand muscles of all participants, showed normal MEP amplitudes and latencies.

MEP amplitudes deviating more than 2 SD from the mean in each experimental condition and single trials contaminated by muscular preactivation were excluded as outliers (1%). Baseline corticospinal excitability was assessed at the beginning and at the end of each experimental session by recording two series of ten MEPs, while participants passively observed a white-colored fixation cross presented on a black background on the computer screen. Comparisons of MEP amplitudes for the two series allowed us to check for any corticospinal excitability change, related to TMS* per se*. The Mann-Whitney* U*-test was used to compare the amplitude of MEPs collected from the two muscles in the baseline trials. For each participant, MEP amplitudes were converted into a proportion of the baseline value. Differences in MEP amplitudes were analyzed nonparametrically with Kruskal-Wallis analysis of variance and Mann-Whitney* U*-test, post hoc tests between patients groups (LHD, RHD) and for comparison of type of movement observed (grasping small object, grasping large object, and static image), and TMS delivery timings. We compared MEP amplitudes of LHD patients, stimulated on the right (contralesional) primary motor cortex (M1), with those of RHD patients, stimulated on the left (contralesional) primary motor cortex (M1).

## 3. Results

### 3.1. Barthel Index (BI) and Functional Independence Measure (FIM) Outcome: LHD versus RHD

Kruskal-Wallis tests were applied to determine whether there was a statistically significant difference in mean BI and FIM scores between groups of patients (RHD, LHD). Results showed that there were no significant differences between groups in BI (*χ* ^2^ = 0.559, df = 1, *P* = 0.455) and FIM (*χ* ^2^ = 0.240, df = 1, *P* = 0.624).

### 3.2. Baseline: TMS Effects on FDI (RHD versus LHD) and ADM (RHD versus LHD)

A preliminary analysis was performed to verify differences depending on videos showing actions performed by either a left or right hand. Those analyses revealed that there were no significant differences associated with videos showing either the left or the right hand grasping the target-object (*P* > 0.1). This variable was thus collapsed during the main analysis.

In order to evaluate whether TMS* per se *could induce any changes in corticospinal excitability in our experimental procedure, we compared mean raw MEP amplitudes during the two baseline blocks, at the beginning and at the end of the experimental session. There was no statistically significant effect for either the contralesional FDI (RHD:* U*(3) = 0.649, *P* = 0.209; LHD:* U*(4) = 0.1702, *P* = 0.340) or the contralesional ADM muscle (RHD:* U*(3) = 0.1440, *P* = 0.579; LHD:* U*(4) = 0.2235, *P* = 0.154) in both groups of patients.

### 3.3. MEPs Related to Observed Actions

Kruskal-Wallis analysis of variance was conducted to evaluate a different involvement of FDI and ADM muscles during observation of grasping actions as a function of group. Both comparisons were significant: FDI (RHD versus LHD): *χ* ^2^ = 6.956, df = 1, *P* = 0.008; ADM (RHD versus LHD): *χ* ^2^ = 6.818, df = 1, *P* = 0.009.

### 3.4. FDI: RHD versus LHD

**Figure 1:**
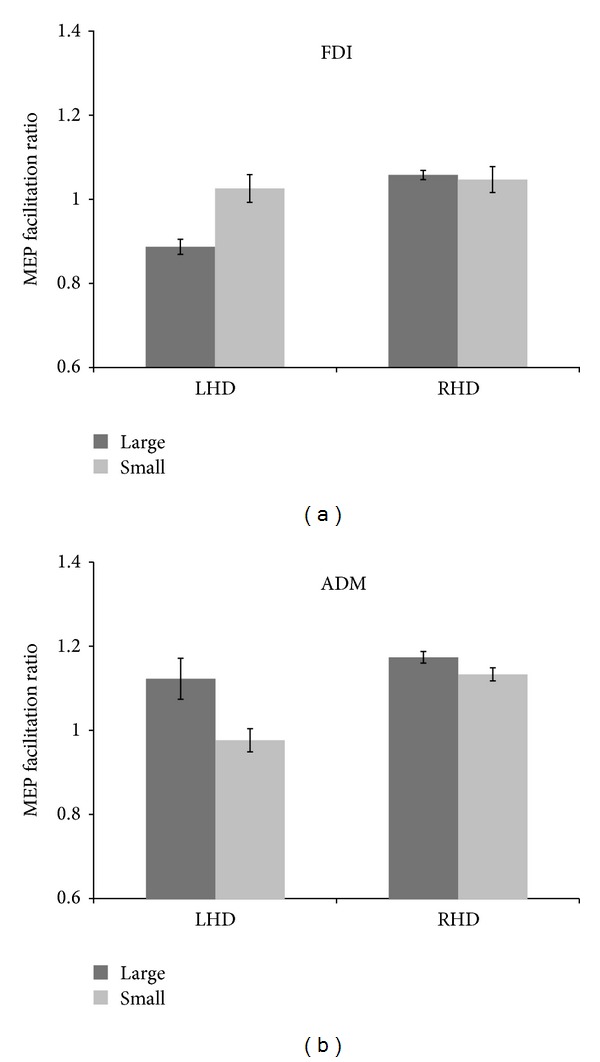
(a) The effects of object sizes on M1 excitability elicited by single-pulse TMS recorded in 9 stroke patients for first dorsal interosseus (FDI) muscle. Motor-evoked potential (MEP) amplitude for object size condition (small or large). Error bars represent the S.E.M. (b) Comparisons of MEP amplitude (*n* = 9) evoked by single-pulse TMS from the abductor digiti minimi (ADM) muscle. Motor-evoked potential (MEP) amplitude for object size condition (small or large). Error bars represent the S.E.M.

Follow-up tests were conducted to evaluate pairwise differences among the three different videoclips, TMS delivery timings, and object size. The results of these tests revealed a significant difference for the FDI muscle in LHD patients (grasping acts to small versus large target-objects). Specifically, we detected a higher facilitation for small objects than for large ones, when patients watched a grasp act and TMS was delivered at the end of the action (*U*(3) = 2.500, *P* = 0.036,* r* = 0.69). On the contrary, in RHD patients, we did not detect any significant difference among the videoclips, TMS delivery timings, and object size (*P* > 0.1) (Figure 1(a)).

### 3.5. ADM: RHD versus LHD

Regarding the ADM muscle, we did not detect any significant modulation in RHD patients (*P* > 0.1). In contrast, in LHD patients, we found a clear modulation of MEP activity, characterized by facilitation when patients observed a grasping act towards a large object rather than towards a small one (*U*(3) = 3.000, *P* = 0.047,* r* = 0.66). Moreover, this modulation was present only for videos showing a grasping movement when TMS was delivered at the beginning or at the end of the grasp act (*U*(3) = 2.000, *P* = 0.028,* r* = 0.73) (Figure 1(b)).

## 4. Discussion

The present study revealed that during the observation of an object-related action, the cortical representation of hand muscles is significantly enhanced in a sample of hemiplegic stroke patients. The cortical excitability changes induced by the type of grasp observed are very similar to those observed during voluntary hand movement in healthy participants.

More specifically, we found that patients who are impaired by left-hemisphere stroke are facilitated on the right (contralesional) M1 during action observation of hand-object interactions. When the action observed was a whole hand grasp (large objects), ADM was facilitated. Conversely, FDI but not ADM was facilitated when the action observed was a precision grip (small objects). In contrast, in patients who had experienced a right hemisphere stroke, left M1 is not facilitated during action observation for both muscles considered (ADM and FDI). Note that all stroke patients did not show any reliable MEP in the two hand muscles recorded, after stimulation of their affected hemisphere.

As previously confirmed in healthy participants, the specific excitability changes of the cortical representation of the muscle involved in the observed movement reflect the fact that motor facilitation is strictly related to the motor strategy adopted by the subject [28, 29]. This suggests that stroke patients often maintain the ability to mentally simulate movements in ways that are consistent with the principles of limb biomechanics. Presumably, at some level in the unimpaired hemisphere there are representations of the limb that remain intact and can be accessed and used to solve motor deficits after stroke.

The pattern of facilitation induced by passive observation of hand-object interactions is different in the nonmotor dominant hemisphere (i.e., right) compared with the motor dominant hemisphere (i.e., left), probably for a different excitatory state of the motor cortex in the right but not in the left hemisphere. This difference could be attributed to a decrease of inhibitory inputs to the M1. This finding is supported by TMS studies on excitability of the M1 inhibitory interneurons. In these studies, a reduced amount of intracortical inhibition in the unaffected hemisphere of stroke patients has been reported [30, 31].

Stroke is a classical model of an unbalanced activity of the two M1s supported by an abnormal stronger interhemispheric communication from the impaired to the intact hemisphere [31]. Converging studies support the notion that the motor system, after damage, generates the best functional motor output, given the anatomical constrains [32, 33]. In cortical lesions involving the primary motor cortex, the unaffected M1 hyperexcitability might be the result of a deregulation of the excitatory/inhibitory circuits, because of the breakdown of the reciprocal connections within the parietofrontal network. The functional consequences of this unaffected M1 hyperexcitability could influence activity-dependent plastic changes.

Interestingly, motor facilitation in right M1 in left hemisphere stroke patients could be explained by the necessity to increase the use of left (unaffected, nondominant) hand by these patients for activities in daily living resulting in a better dexterity of the left hand and a possible shift towards right hemisphere motor dominance. By contrast, in patients with right hemisphere impairment we did not detect modifications in corticospinal excitability during any of the grasp type observed, plausibly because the dominant hand (i.e., left) is unaffected and already involved in daily activities.

Furthermore, right M1 facilitatory modulation in stroke patients seems to be linked to the relative motor activation produced by transitive (i.e., goal directed) versus intransitive behaviors [7, 34]. According to this view, the neural activity during observation is attributable to the covert generation of a motor command and we observe congruent neural activity during observation, because the visual goal, and thus the motor command generated, is the same as during active movement. In the current study we extended findings of previous studies [14–17] by showing that also during movement observation, corticospinal excitability depends on task complexity (the use of a whole hand versus precision grips). This implies that activity of motor cortical areas is sensitive to the context in which the movement is performed.

One limitation of the current study is the use of a relatively simple task with low ecological value (i.e., videos showing an isometric task using a power versus a precision grip). In addition, in the present study we used an egocentric perspective; it is known that the MEP facilitation induced by observation is larger from an egocentric perspective than from an allocentric perspective. Another potential limitation is that the two groups of patients were not matched for stroke location. Nonetheless, we were able to elicit MEPs in contralesional hand muscles, indicating that their corticomotor pathways were similarly preserved. This indicates that differences between groups in MEP facilitation during action observation are not due to differences in the extent of subcortical damage to the corticospinal pathway.

The results of the present study support the idea of using action observation to prime the motor system by showing that specific motor plans in damaged motor circuits are activated by action observation after stroke. In particular we observed a muscle-specific motor facilitation induced by observation of simple everyday actions, at least in left-motor dominant hemisphere stroke patients.

Different involvement of FDI and ADM hand muscles reflect the muscular activity characterizing the type of grasp observed. When the target was a large object, ADM facilitation was increased while FDI activation was decreased. Conversely, FDI facilitation was more pronounced when the target was a small object than when the target was a large object. As reflected in the excitability level of relevant muscle representations, the corticospinal system seems to be able to store an internal representation of motor outputs and it can rapidly adapt its state to generate the most appropriate motor command.

The current results confirm the existence of a partially lateralized network that represents manual skills [35, 36]. This network is activated after a stroke, when patients are observing movements performed by a demonstrator's hand. These findings support the idea that this network permits patients to mentally simulate movements in the hand that will become dominant.

A key issue in rehabilitation is whether interventions should be aimed at treating the underlying impairment (i.e., restoring the lost function) or they should seek to provide patients with strategies that enable them to compensate for their impairments. It is promising that after stroke, action representations of the paretic limb are preserved and may be activated by action observation. Retraining approaches to rehabilitation make the assumption that practicing a particular cognitive function through tasks and exercises will enable that function to return in a more or less normal fashion. This form of rehabilitation could be a powerful treatment approach for patients with hemiplegic stroke.

## Figures and Tables

**Table 1:** Demographic of patients.

Subjects	Sex	Age	Education	Lesion side	Years poststroke^a^	Barthel index (BI)	FIM
1	M	58	8	L	4.5	70	90
2	M	46	8	R	5.2	80	80
3	M	54	11	R	7.8	90	68
4	M	70	8	R	5.2	65	71
5	M	69	5	R	3.4	10	53
6	M	59	8	L	6.6	65	69
7	M	67	13	L	4.5	40	58
8	M	37	11	L	3.6	65	70
9	M	65	11	L	3.3	60	74

BI: Barthel index; FIM: functional independence measure.

^a^Years poststroke are calculated as time elapsed between stroke onset and day of data collection.
